# A Hybrid Particle-Flow CFD Modeling Approach in Truncated Hepatic Arterial Trees for Liver Radioembolization: A Patient-specific Case Study

**DOI:** 10.3389/fbioe.2022.914979

**Published:** 2022-05-30

**Authors:** Tim Bomberna, Saar Vermijs, Maryse Lejoly, Chris Verslype, Lawrence Bonne, Geert Maleux, Charlotte Debbaut

**Affiliations:** ^1^ IBiTech-Biommeda, Department of Electronics and Information Systems, Ghent University, Ghent, Belgium; ^2^ Cancer Research Institute Ghent, Ghent University, Ghent, Belgium; ^3^ Department of Radiology and Medical Imaging, Ghent University Hospital and Ghent University, Ghent, Belgium; ^4^ Department of Clinical Digestive Oncology, University Hospitals Leuven, Leuven, Belgium; ^5^ Department of Radiology, University Hospitals Leuven, Leuven, Belgium; ^6^ Department of Imaging and Pathology, KU Leuven, Leuven, Belgium

**Keywords:** computational fluid dynamics, hepatocellular carcinoma, liver radioembolization, drug delivery & targeting, *in silico* medicine, pretreatment planning, virtual twins

## Abstract

Hepatocellular carcinoma (HCC) is the most common form of primary liver cancer. At its intermediate, unresectable stage, HCC is typically treated by local injection of embolizing microspheres in the hepatic arteries to selectively damage tumor tissue. Interestingly, computational fluid dynamics (CFD) has been applied increasingly to elucidate the impact of clinically variable parameters, such as injection location, on the downstream particle distribution. This study aims to reduce the computational cost of such CFD approaches by introducing a novel truncation algorithm to simplify hepatic arterial trees, and a hybrid particle-flow modeling approach which only models particles in the first few bifurcations. A patient-specific hepatic arterial geometry was pruned at three different levels, resulting in three trees: Geometry 1 (48 outlets), Geometry 2 (38 outlets), and Geometry 3 (17 outlets). In each geometry, 1 planar injection and 3 catheter injections (each with different tip locations) were performed. For the truncated geometries, it was assumed that, downstream of the truncated outlets, particles distributed themselves proportional to the blood flow. This allowed to compare the particle distribution in all 48 “outlets” for each geometry. For the planar injections, the median difference in outlet-specific particle distribution between Geometry 1 and 3 was 0.21%; while the median difference between outlet-specific flow and particle distribution in Geometry 1 was 0.40%. Comparing catheter injections, the maximum median difference in particle distribution between Geometry 1 and 3 was 0.24%, while the maximum median difference between particle and flow distribution was 0.62%. The results suggest that the hepatic arterial tree might be reliably truncated to estimate the particle distribution in the full-complexity tree. In the resulting hybrid particle-flow model, explicit particle modeling was only deemed necessary in the first few bifurcations of the arterial tree. Interestingly, using flow distribution as a surrogate for particle distribution in the entire tree was considerably less accurate than using the hybrid model, although the difference was much higher for catheter injections than for planar injections. Future work should focus on replicating and experimentally validating these results in more patient-specific geometries.

## 1 Introduction

Global cancer incidence is expected to double by 2035, putting strain on healthcare systems and healthcare financing worldwide, and increasing the need for efficient and effective cancer care pathways (Prager et al., 2018). Hepatocellular carcinoma (HCC), the most common form of primary liver cancer, is one of the most severe cancers worldwide, ranking sixth in diagnosis and fourth in mortality around the globe (Philips et al., 2021). Major risk factors for HCC include Hepatitis B and C infection (expected to decrease because of widely promoted vaccination programs), excessive alcohol consumption, the mycotoxin aflatoxin B1 (expected to increase because of climate change), non-alcoholic fatty liver disease, diabetes and obesity (McGlynn et al., 2021). At its intermediate, unresectable stage, HCC is typically treated by transarterial therapies such as transarterial chemo- and radioembolization (TACE and TARE). For these transarterial therapies, catheters are retrogradely advanced *via* the femoral artery towards the hepatic arterial bed, where damaging microspheres are locally injected to selectively damage tumor tissue. In the case of TACE, these microspheres damage the tumor tissue through a combined chemotherapeutic and embolic effect. In the case of internal radiotherapy by means of TARE, the microspheres are typically smaller in diameter, and tumor tissue is damaged mainly through the spread of radioactivity (Salem and Lewandowski, 2013). Up to now, no clear evidence has emerged for superiority of TACE over TARE, or vice versa (Facciorusso et al., 2016). With regards to side-effects, the post-embolization syndrome is much less common in TARE because the endpoint of injection is not total flow obstruction, contrary to TACE (Facciorusso et al., 2016). However, TARE procedures are complicated by the possible delivery of higher-than-acceptable radioactive doses to the surrounding healthy parenchyma and the lungs (Ahmadzadehfar et al., 2010). Therefore, a pre-treatment SPECT scan with injection of Technetium-99m-macroaggregated albumin (Tc-99m-MAA) microparticles is usually performed before TARE to estimate the intra- and extrahepatic microsphere spread (Ahmadzadehfar et al., 2014). For both therapies, minimizing (or, preferably, eliminating) the delivery of microspheres to the surrounding healthy parenchyma is of crucial importance to obtain optimal treatment outcomes. Clinically, the execution of these transarterial therapies depends on the implementation of a wide range of variable parameters, such as the microparticle type, the injection device, etc. Focusing on TARE, two Yttrium-90 microsphere agents are commercially available: the resin-based SIR-Spheres (Sirtex Medical, Australia) and the glass-based TheraSpheres (MDS Nordion, Canada). These microspheres differ in several biophysical properties, such as diameter range (20–60 μm for SIR-Spheres vs. 20–30 μm for TheraSpheres), density (1,600 kg/m³ vs. 3,200 kg/m³) and activity per particle (50 vs. 2,500 Bq) (Ahmadzadehfar et al., 2010). Importantly, the pre-treatment Tc-99m-MAA microparticles also have different biophysical characteristics (mean diameter: 15 μm, density: 1,100 kg/m³) compared to the afore-mentioned treatment particles, which may induce a discrepancy in pre-treatment dose prediction and actual treatment dose delivery (Wondergem et al., 2013). For example, Jiang et al. (2012) compared segmental activity for the pre-treatment injection of Tc-99m-MAA microparticles and post-treatment SPECT imaging of Yttrium-90 radiotracers and found significant differences in 31 out of 81 treatments. While 24 of the 31 discrepancies could be explained by a slight shift in catheter position, 5 differences could not explained, underlining both the key impact of catheter position and the variability of these therapies (Jiang et al., 2012). Additionally, other radiotracers than Yttrium-90 can be used, such as Holmium (Reinders et al., 2019). QuiremSpheres (Terumo, Japan), embedded with Holmium, have the property of being highly paramagnetic, meaning that they can be visualized *in vivo* using MRI. Herein, the added value is that QuiremSpheres can be used both for the pre-treatment scout dose and the actual treatment, minimizing previous discrepancies in microparticle characteristics between treatment and pre-treatment (Roosen et al., 2021). Next, with regard to injection devices, several commercial catheter types exist, such as the standard microcatheter (offered by several companies such as Terumo (Japan), Guerbet (France), Boston Scientific (United States), etc.), the balloon-occluding catheter (e.g., Occlusafe Temporary Occlusion Balloon Catheter (Terumo, Japan); Sniper Balloon Microcatheter (Embolx, United States)), antireflux catheters (e.g., Surefire Infusion System, Surefire Medical, United States), etc. Typically, transarterial therapies are envisioned as flow-directed therapies, meaning that they rely on tumor-directed flow to carry microspheres downstream to the target tissue. However, alternative catheter designs such as the balloon-occluding or antireflux catheters obstruct the flow and significantly alter the pressure and flow patterns in the downstream vascular compartment, which may impact the microsphere transport (Rose et al., 2019). Similarly, other clinically variable parameters, such as infusion speed and catheter tip position may also play a significant role in downstream microparticle distribution (Aramburu et al., 2017). Importantly, the reported mean survival time after TACE ranges from 3.4 to 31 months (median of 14 months) in prospective studies (Raoul et al., 2011). This range in survival time could be partly explained by the lack of procedure standardization, or different executions of transarterial therapies. However, it is currently unclear to which extent the above-mentioned heterogeneities in treatment execution impact the microparticle flow, downstream distribution and, by extent, the treatment response. Interestingly, Computational fluid dynamics (CFD) outline a series of numerical modeling techniques to solve problems related to fluid flow. In the past decade, CFD has increasingly been used to model blood flow and microparticle transport in the hepatic arteries to mimic transarterial therapies such as TACE and TARE (Basciano et al., 2010; Kennedy et al., 2010; Childress et al., 2012; Aramburu et al., 2016a; Aramburu et al., 2017; Bomberna et al., 2021). Importantly, CFD has already shown to be a useful tool in the evaluation of the role of several clinically variable parameters on the microsphere distribution in patient-specific geometries. For example, Basciano et al. (2010) investigated the impact of injection timing and particle properties on microsphere release, noting that the discrepancy in physical properties of SIR-Spheres and TheraSpheres mattered more during the decelerating section of the cardiac inflow cycle, because of increasing importance of inertial effects. Inspecting the impact of catheter type, Aramburu et al. (2016a) compared the standard end-hole microcatheter with a wide-tip antireflux catheter, discovering that the extent of catheter tip greatly impacted the streamline pattern of the infusion fluid (modelled as blood in their study) upon catheter exit. Over time, it has been shown multiple times that the axial and cross-sectional catheter position have a considerable impact on the microsphere distribution in both idealized geometries (Basciano et al., 2010; Kennedy et al., 2010) and in patient-specific geometries as shown in our previous work (Bomberna et al., 2021). Additionally, Aramburu et al. have indicated that not only catheter position plays an impact, but that also the microcatheter tip orientation and catheter distal direction are parameters of importance (Aramburu et al., 2016b; Aramburu et al., 2017). These studies all highlight how CFD may be used as an investigative tool. Importantly, the patient-specific nature of each clinical case should be stressed. For example, while the impact of catheter position on microsphere distribution is clear from a range of studies, the optimal catheter position cannot be determined on a general basis. This is partly because many hepatic arterial anatomic variants exist, with the standard normal anatomy (according to Michel’s classification (Michels, 1966)) occurring in only 55%–76% of patients (Favelier et al., 2015). Additionally, the geometry may be severely impacted in cancer patients, while the degree of cancer burden and location may also vary significantly between patients. Hence, CFD can assist in transforming the current treatment execution into a patient-specific workflow by 1) pre-emptively estimating the sensitivity of the microparticle distribution to the implementation of the aforementioned treatment parameters, 2) identifying the most impactful parameters, and 3) optimizing these parameters in the patient-specific setting. Similar to therapy planning and optimization, Roncali et al. (2020) have forwarded a CFD approach for personalized dosimetry of TARE (although microparticle behavior was not explicitly modelled in that approach), showing the many possible applications of CFD techniques to optimize the execution and pre-treatment planning these transarterial therapies. One of the key problems regarding these CFD techniques is that large-scale validation is still missing. Still, Antón et al. (2021) have performed a proof-of-concept *in vivo* validation study of their computational approach by focusing on three patients, showing a considerable agreement between the *in vivo* and computational particle distribution. Similarly, *in vitro* validation in 3D-printed patient-specific hepatic arterial circuits has been used to validate particle distribution given a set of outlet flows during our previous work (Bomberna et al., 2021). These preliminary *in vivo* and *in vitro* validations show the potency of CFD techniques, but need to be significantly expanded before clinical implementation. Another important factor with regards to clinical application is the computational complexity of these CFD simulations, since large computational times may negatively impact the potential of transferring this technology to the clinical setting. A clear and uniform workflow for these CFD simulations—including standards for the definition of hepatic arterial geometries, and inlet and outlet boundary conditions, which currently vary between computational approaches—is still lacking (Bomberna and Debbaut, 2021). Therefore, future efforts should prioritize outlining a time-efficient, reliable and validated CFD methodology for patient-specific therapy planning and dosimetry. Hence, this study aims to reduce the computational cost of these CFD approaches, while outlining a clear methodology to define the hepatic arterial geometry and outlet boundary conditions. The main goal is to evaluate whether the particle distribution in a complex patient-specific geometry can be estimated by modeling the particle distribution in a truncated, simplified geometry, and assuming that the particles downstream of the truncated outlets distribute themselves proportional to the blood flow. This hybrid “particle-flow” model should significantly reduce the complexity of the computational approach, compared to explicit particle modeling in the entire arterial geometry. Additionally, the fitness of the flow distribution (i.e., no explicit particle modeling) as a surrogate for particle distribution will also be considered. To define how the hepatic arterial geometry may be truncated, a tumor-based pruning algorithm is developed and evaluated for a patient-specific case. Previously, Lertxundi et al. (2021) introduced a segment-based pruning algorithm to simplify patient-specific arterial geometries, comparing truncated versions of 3 patient-specific geometries (with 1 catheter tip location for 2 geometries and 2 catheter tip locations for the other geometry). However, the alternative pruning algorithm presented here goes beyond the state-of-the-art by using a novel tumor region growing model. Additionally, this algorithm can also inform outlet boundary conditions, identify the major arterial feeders of the tumor, and estimate the total dose delivered to the tumor. During this study, the hybrid particle-flow models for truncated hepatic arterial geometries will be evaluated for one planar injection (where particles are released over the entire vessel cross-section) and three catheter injections (where particles are only released from the catheter tip) for one patient-specific case.

## 2 Materials and Methods

To evaluate the hybrid particle-flow model in truncated arterial geometries, the details regarding the reconstruction and discretization of the full-complexity hepatic arterial geometry and the truncation algorithm are presented (Section 2.1). Next, the mathematical modeling behind the particle-flow model (including inlet and outlet boundary conditions) is explained (Section 2.2). Finally, several metrics are introduced which will help compare the hybrid particle-flow model against the particle distribution in the full-complexity geometry (Section 2.3).

### 2.1 Geometry Development and Discretization

#### 2.1.1 Study Design

In Table 1, an overview of the numerical simulations in this study are presented. In summary, this study considers the same set of simulations for a full-complexity hepatic arterial geometry (Geometry 1) and two arterial geometries with different levels of truncation (Geometry 2 & 3; see Section 2.1.3): one planar injection at a specified axial location (Sim. 1–3 in Table 1; see Section 2.2.2.1), and three catheter injections at specific cross-sectional locations on this plane (Sim. 4–12 in Table 1; see Section 2.2.2.1).

**TABLE 1:** Study design giving an overview of all simulations and their corresponding geometry, injection type and location.

Simulation (Sim.)	Geometry	Injection	Injection location
Sim. 1	Geometry 1	Planar	Axial Location 1
Sim. 2	Geometry 2	Planar	Axial Location 1
Sim. 3	Geometry 3	Planar	Axial Location 1
Sim. 4	Geometry 1	Catheter	Inlet: Cross-Sectional Location 1
Sim. 5	Geometry 2	Catheter	Inlet: Cross-Sectional Location 1
Sim. 6	Geometry 3	Catheter	Inlet: Cross-Sectional Location 1
Sim. 7	Geometry 1	Catheter	Inlet: Cross-Sectional Location 2
Sim. 8	Geometry 2	Catheter	Inlet: Cross-Sectional Location 2
Sim. 9	Geometry 3	Catheter	Inlet: Cross-Sectional Location 2
Sim. 10	Geometry 1	Catheter	Inlet: Cross-Sectional Location 3
Sim. 11	Geometry 2	Catheter	Inlet: Cross-Sectional Location 3
Sim. 12	Geometry 3	Catheter	Inlet: Cross-Sectional Location 3

#### 2.1.2 Baseline Geometry and Tissue-Perfusion Modeling

**FIGURE 1:**
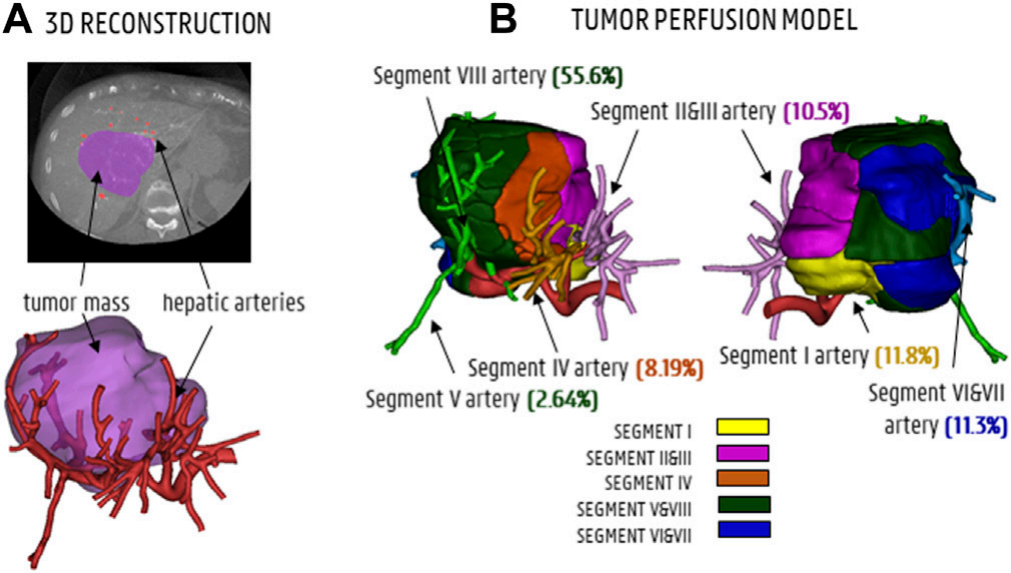
**(A)** CT-scan slice and 3D reconstruction showing the tumor mass (purple) and hepatic arteries (red). **(B)** The tumor perfusion model shows to which degree the segmental arteries I, II and III, IV, V and VIII and VI and VII contribute to tumor perfusion (TPPs in %).

As approved by the Ethical Committee of the University Hospitals Leuven (UZ Leuven, Belgium), a patient-specific CT-image dataset of the hepatic arterial vasculature of an HCC patient was obtained by scanning the patient with a conebeam CT scanner (Philips Medical Systems, Netherlands) while intra-arterially injecting contrast agent into the left and right branches of the proper hepatic artery. The hepatic arteries were segmented in Mimics (Materialise, Belgium) based on the contrast difference between the arterial and venous trees in the arterial phase. A large tumor nodule (estimated volume: 310 ml) was identified. 3D reconstructions of the tumor mass and the hepatic arterial tree (with 1 inlet at the proper hepatic artery level and 48 outlets) can be found in Figure 1A. For CFD purposes, the division of the hepatic artery outlets into tumor-perfusing outlets and healthy tissue-perfusing outlets is crucial in order to know which vessels should be targeted for the envisioned treatment. Therefore, the tumor perfusion percentage (TPP) of each outlet was determined as the percentage of the tumor volume perfused by each outlet (e.g., 0% for solely healthy tissue-perfusing outlets). To calculate the TPP of the 48 outlets, an in-house developed region growing model was used (Vermijs et al., 2021). First, the centerlines of the hepatic arterial trees were determined using the open-source Vascular Modelling Toolkit (vmtk.org). For each artery outlet segment, the centerline points between the final bifurcation and the outlet surface were labelled as seed points. Second, the hepatic arteries and tumor mass were included in a voxelated bounding box, consisting of 100 × 100 × 100 cubic voxels (with a ∼1.2 10^−3^ m edge length). During region growing, voxels were added in the six orthogonal directions starting from the seed points for each segment, until all voxels within the tumor were assigned to one of the outlets. Region growing occurred simultaneously for all branches. As a result, each arterial branch was associated with a certain perfusion zone of the tumor (and, by extension, a certain perfused volume of tumor tissue). Finally, the tumor volume perfused by each outlet was divided by the total tumor volume, giving the TPP. In Figure 1B, the tumoral mass is divided in regions to show how the different segmental arteries contribute to tumor perfusion according to the tumor region growing model. Generally, the liver can be divided in eight segments according to Couinaud’s classification criteria (Coinaud, 1957). For the color code in Figure 1B, the liver is anatomically divided into five sections based on these segments: the caudate lobe (Segment I, in yellow), the left lateral section (Segments II and III, in purple), the medial section (Segment IV, in orange), the right anterior section (Segments V and VIII, in green), and the right posterior section (Segments VI and VII, in blue). For the TPPs in Figure 1B, the segmental arteries are colored according to the same color code, but the annotations for Segments V and VIII are split because of the considerable difference in tumor perfusion (2.64% vs. 55.6%; the Segment V artery clearly points away from the tumor, while the Segment VIII artery points towards the tumor). ‬‬‬‬‬‬‬‬‬‬‬‬‬‬‬‬‬‬‬‬‬

#### 2.1.3 Truncation Algorithm

**FIGURE 2:**
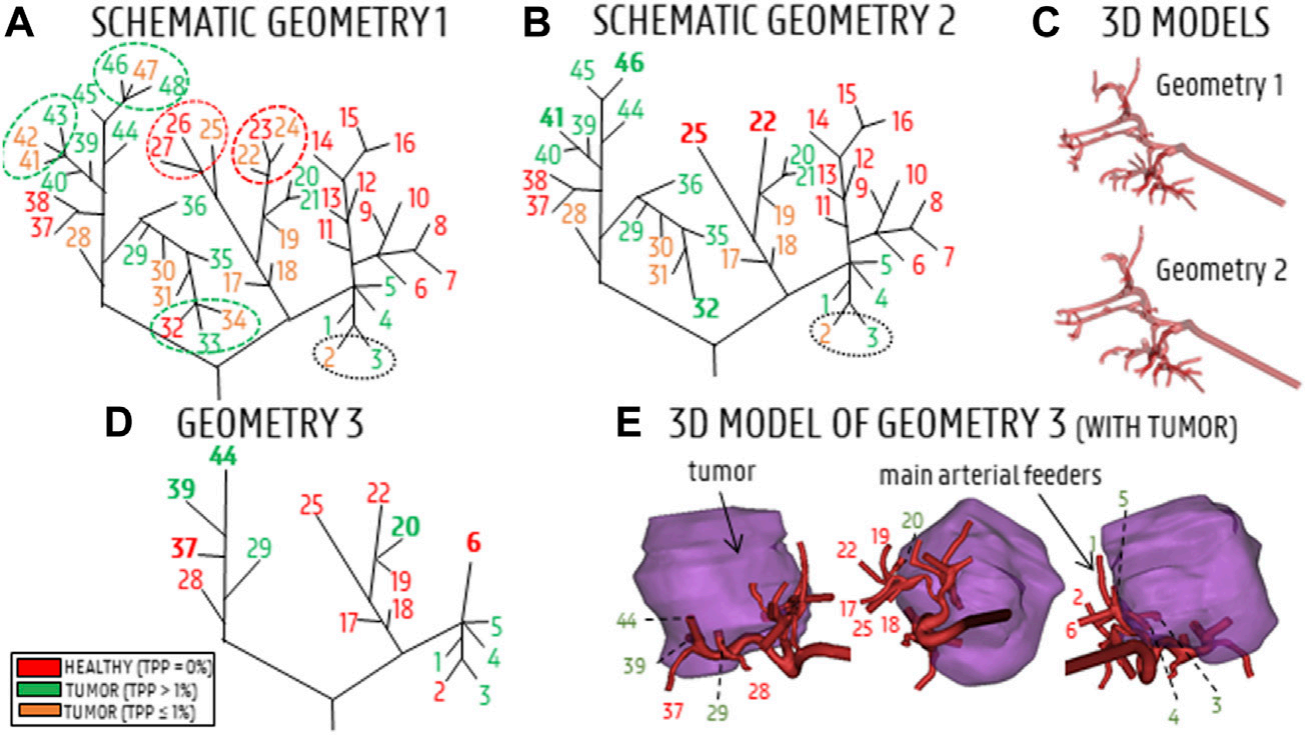
**(A)** Schematic of Geometry 1. Red branches indicate healthy branches (TPP = 0%), orange branches indicate branches that perfuse a small fraction of the tumor (TPP≤1%), green branches indicate branches that perfuse a significant portion of tumor tissue (TPP>1%). Ellipses indicate which branches of Geometry 1 were pruned. **(B)** Schematic of Geometry 2; bold indices indicate where arteries were pruned. **(C)** 3D reconstructions of Geometries 1 and 2. **(D)** Schematic of Geometry 3, with the main arterial feeders denoted in green and the healthy-perfusing branches denoted in red. **(E)** 3D model of Geometry 3 (3 views), showing the relative position of the 17 outlets with respect to the tumor mass.

The baseline hepatic arterial tree was imported into ANSYS SpaceClaim (Ansys, United States) and manually reconstructed to generate a solid model. During the truncation process (see below), the arterial tree was pruned at three different levels, generating three solid arterial tree models with varying degrees of complexity. The full-complexity hepatic arterial tree was denoted as Geometry 1 (48 outlets, no pruning, shown in Figure 1A, with schematic illustration in Figure 2A). The diameters of these 48 outlets are given in Table 2 (estimations were made using the Fit Centerline and Best Fit Diameter tools in Mimics (Materialise, Belgium)). The truncation process is explained in detail below. First, distal bifurcations containing outlets with TPPs ≤1% (orange branches in Figure 2A) were pruned. If the total TPP of the bifurcations containing one or more of these orange outlets was >1%, the resulting outlet (i.e., after pruning) was denoted as “tumor” (green dotted ellipses in Figure 2A); otherwise, the resulting outlet was denoted as “healthy” (red dotted ellipses in Figure 2A). Outlets which where impossible to prune due to the lack of cutting space (i.e., when bifurcation points were located so close to each other that a proper cut could not be made) were preserved (e.g., branches 2 and 3 in Figure 2A,B). In total, the total number of outlets was reduced from 48 to 38 outlets in this step. After pruning, some outlets were located so close to the preceding bifurcation (<5 mm distance on the centerline) that fluid flow into these pruned outlets would not be properly developed. Therefore, these outlets were artificially extruded to a total length of 20 mm (e.g., branches 22 and 25 in Figure 2B). Hence, the first part of the truncation process resulted in the second solid model, denoted as Geometry 2 (38 outlets, schematic in Figure 2B). The numbering of the resulting outlets after pruning was done according to the lowest number of the group of outlets that were pruned (i.e., if outlets 22, 23 and 24 were pruned into a single outlet, the resulting outlet was given the number 22.) In the next step of the truncation process, bifurcations containing outlets which perfused the same tissue type (either “healthy” or “tumor”) were pruned (green and red circles in Figure 2B); bifurcations which contained both were not simplified (e.g., the bifurcation consisting of branches 37–38 and 39–46 in Figure 2B). The few remaining outlets with TPP <1% (orange branches in Figure 2B) were pruned according to the same methodology as before: if the total bifurcation to which the outlet belonged had a TPP>1%, the resulting outlet was considered “tumor” (e.g., the case for branches 30–31, which were pruned and resulted in outlet 29 in Figure 2D); if the TPP<1%, it was considered “healthy”. Similarly, pruned outlets that were too short (<5 mm on the centerline) were extended to a total length of 20 mm (e.g., branch 6 and 44 in Figure 2D). The second step of the truncation process resulted in the third and final solid model, denoted as Geometry 3 (17 outlets, Figure 2C). In Geometry 3, the remaining branches with TPPs ≤1% - which could not be pruned in steps one or two - are considered “tumor” if the tumor flow contribution in that branch is >50% of the total flow through that branch (considering that each of these branches perfuses both the tumor and a fraction of the healthy tissue), and considered as “healthy” if the tumor flow contribution is <50%. A detailed explanation of how the healthy and tumor flow contributions were determined is outlined in Section 2.2.2.2. As a result, 8 out of 17 branches in Geometry 3 (i.e., branches 1, 3, 4, 5, 20, 29, 39, and 44) are considered as “tumor” (green branches in Figure 2D); these are the main arterial feeders that feed 98% of the tumor tissue. Additionally, in these feeders, the tumor flow contribution ranges from 48 to 94% of the total flow through the feeders. In Figure 2E, three 2D-views of the 3D models of the hepatic arteries of Geometry 3 are shown, indicating the relative position of the main arterial feeders to the tumor mass. It can be seen that the main arterial feeders are either pointing towards the tumor mass, or are located inside the tumor mass. Importantly, the truncation algorithm did not allow for truncation of the two most proximal bifurcation levels in the arterial tree, since it is assumed that these bifurcations could play an important role in downstream particle distribution.

**TABLE 2:** Overview of the sizes and flow fractions for the 48 outlets of Geometry 1.

Outlet	Diameter [10^−3^ m]	Flow fraction [%]
1	3.58	4.07
2	3.23	0.79
3	4.94	4.37
4	4.12	3.45
5	3.39	2.99
6	3.31	0.69
7	3.05	0.62
8	3.08	0.62
9	3.35	0.62
10	3.33	0.62
11	3.6	0.34
12	3.22	0.11
13	3.36	0.11
14	3.26	0.11
15	3.10	1.24
16	2.77	1.24
17	3.25	3.66
18	3.2	1.80
19	3.35	0.33
20	4.08	3.05
21	3.26	0.84
22	3.26	0.23
23	2.29	0.11
24	3.34	0.12
25	3.11	0.50
26	3.48	0.22
27	3.01	0.22
28	3.32	3.63
29	3.4	2.60
30	2.76	1.33
31	3.01	0.26
32	3.02	0.07
33	2.74	0.77
34	2.94	0.07
35	3.35	0.97
36	4.41	8.94
37	3.73	0.82
38	2.85	0.82
39	3.33	13.32
40	2.75	6.03
41	3.25	3.39
42	3.08	2.99
43	2.92	6.00
44	3.69	2.59
45	3.55	3.95
46	3.01	4.32
47	3.81	0.59
48	2.54	3.47

#### 2.1.4 Catheter Modeling

**FIGURE 3:**
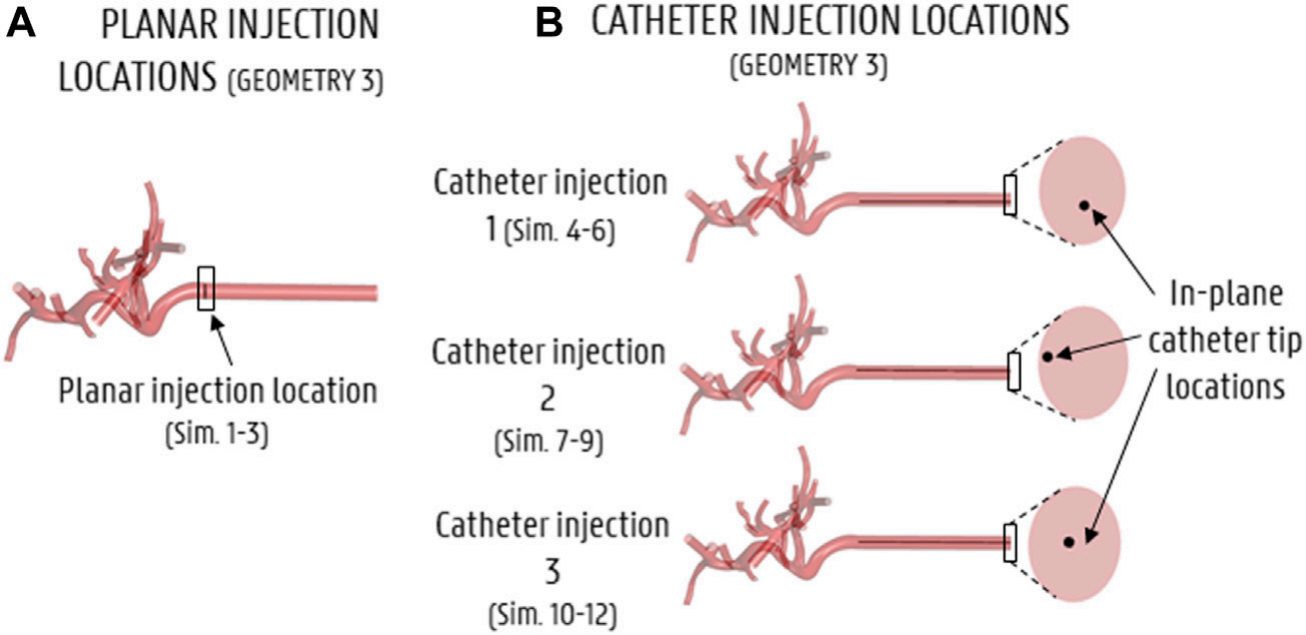
Particle injection locations for all simulations (shown here for Geometry 3). **(A)** Axial injection location for the planar injection, **(B)** axial and in-plane catheter tip locations for the 3 catheter injections.

The truncation process resulted in three arterial geometries of differing complexity, with the number of outlets varying from 17 (Geometry 3) to 48 (Geometry 1). For each geometry, the inlet was extruded by 80 mm in SpaceClaim (Ansys, United States). This was done to account for the entrance length (estimated as ∼80 mm) so that a computationally straightforward, uniform velocity could be applied at the extruded inlet, and a physiological parabolic-like velocity profile would develop before the “true” inlet of the geometry. Next, four versions of the solid model of each geometry were made: three with catheters embedded in the lumen of the proper hepatic artery at different cross-sectional positions (see Section 2.2.2.1 and Figure 3B), and one without a catheter (see Section 2.2.2.1 and Figure 3A). The catheter was modelled as a thin-walled, straight tube, with a total length of 80 mm and a representative inner diameter of 0.7 mm. In total, 12 solid models were made, totaling 3 planar injections in Geometries 1–3 (Sim. 1–3 in Table 1) and 9 catheter injections in Geometries 1–3 (Sim. 4–12).

#### 2.1.5 Geometry Discretization

The twelve solid models were imported into ANSYS Fluent Meshing (Ansys, United States). For all geometries, the minimum and maximum size of the surface mesh elements were set as 8·10^−6^ m and 3.5·10^−4^ m, respectively, with a growth rate of 1.2. For the solid models without catheters embedded in the lumen, the size of the tetrahedral volume elements of the arterial body was set to 3·10^−4^ m (which was determined through mesh sensitivity studies). Three layers of prism elements were enabled near the walls to better capture the near-wall fluid behavior, with a growth rate of 1.2. This resulted in meshes of 6.68·10^6^, 6.19·10^6^, and 3.97·10^6^ elements for Geometry 1, 2 and 3, respectively (Sim. 1–3). For the solid models with catheters, the arterial body sizing was kept the same, while the tetrahedral element body sizing of the catheter was set at 7·10^−5^ m (determined through mesh sensitivity studies). The surface mesh of the catheter inlet was sized at 3·10^−5^ m. Three inflation layers of prism elements near the arterial and catheter walls were also enabled. For Geometry 1, the three catheter models resulted in meshes of 9.74·10^6^, 9.59·10^6^, and 9.73·10^6^ elements (Sim. 4, 7, 10, respectively). For Geometry 2, the three meshes consisted of 9.08·10^6^, 8.94·10^6^ and 10.3·10^6^ elements (Sim. 5, 8, 11, respectively); for Geometry 3, the three meshes contained 6.45·10^6^, 6.32·10^6^ and 6.45·10^6^ elements (Sim. 6, 9, 12). Next, the twelve meshes were imported into Fluent (Ansys, United States) to model the flow and microparticle behavior.

### 2.2 Numerical Model

#### 2.2.1 Multiphysics Flow and Microparticle Model

To model the flow and microparticle distribution in the hepatic arterial geometries, a multiphase approach was employed which considers both the continuous phase (blood) and the discrete phase (microparticles). The governing equations of conservation of mass and momentum are given in Eq. 1 (where $\vec{\text{u}}$ [m/s] is the velocity vector) and Eq. 2 (where ρ [kg/m³] is the density of the fluid, $\bar{\tau }$ is the shear stress tensor, and $\vec{\text{f}}$ [N] are the forces acting on the fluid), respectively:

$$\nabla \cdot \vec{\text{u}}=0$$ (1)

$$\text{ρ}\left(\frac{\partial \vec{\text{u}}}{\partial \text{t}}+\left(\vec{\text{u}}\cdot \vec{\nabla }\right)\vec{\text{u}}\right)=-\nabla \text{p}+\nabla \cdot \bar{\tau }+\vec{\text{f}}$$ (2)

Blood is modeled as an incompressible, shear-thinning fluid with a density of 1,060 kg/m³. Generally, as shown in Eq. 3, shear stress depends on the blood velocity and apparent blood viscosity ( $\text{μ}\left(\dot{\text{γ}}\right)$ ) [kg/(m ⋅ s)], which depends on the shear rate ( $\dot{\text{γ}}$ [s^-1^]). The viscosity of blood is modelled with a simplified Quemada model (Buchanan et al., 2000), which considers that viscosity depends on the hematocrit value and shear rate. According to the simplified Quemada model, viscosity decreases with increasing shear rate, but is assumed equal to ${\text{μ}}_{0}\;$ (3.09·10^–3^ kg/(m ⋅ s)) for higher shear rates for the sake of computational simplicity (as can be seen in Eq. 4, where ${\text{μ}}_{\infty }$ [kg/(m ⋅ s)] is the asymptotic viscosity (implemented here as 2.65·10^–3^ kg/(m ⋅ s)), ${\text{τ}}_{0}$ [Pa] is the apparent yield shear stress (4.36·10^–3^ Pa), and λ [1/s] is the shear stress modifier (2.18·10^–2^ 1/s). Shear rate can be calculated according to Eq. 5.

$$\bar{\tau }=\text{μ}\left(\dot{\text{γ}}\right)\left[\nabla \vec{\text{u}}+{\left(\nabla \vec{\text{u}}\right)}^{\text{T}}\right]$$ (3)

$$\text{μ}\left(\dot{\text{γ}}\right)=\max\left\{{\text{μ}}_{0},{\left(\sqrt{{\text{μ}}_{\infty }}+\frac{\sqrt{{\text{τ}}_{0}}}{\sqrt{\text{λ}}+\sqrt{\dot{\text{γ}}}}\right)}^{2}\right\}$$ (4)

$$\dot{\text{γ}}=\sqrt{\nabla \vec{\text{u}}\left[\nabla \vec{\text{u}}+{\left(\nabla \vec{\text{u}}\right)}^{\text{T}}\right]}$$ (5)

Microparticles are modelled as inert spheres with a diameter $\left({\text{d}}_{\text{p}}\right)$ of 40·10^–6^ m and a density $\left({\text{ρ}}_{\text{p}}\right)$ of 1,600 kg/m³, similar to SIR-spheres. The microparticle trajectories throughout the hepatic arterial geometries can be calculated by integrating the force balance (which equals the product of particle mass $\left(m_{\text{p}}\left[kg\right]\right)$ and particle acceleration ( $\frac{\text{d}\vec{{\text{u}}_{\text{p}}}}{\text{dt}}\;\left[m/s^{2}\right]$ ), as given by Newton’s second law in Eq. 6) twice:

$$m_{\text{p}}\frac{\text{d}\vec{{\text{u}}_{\text{p}}}}{\text{dt}}=\vec{{\text{F}}_{\text{G}}}+\vec{{\text{F}}_{\text{D}}}+\vec{{\text{F}}_{\text{P}}\;}+\vec{{\text{F}}_{\text{V}}\;}$$ (6)

where the following forces acting on the microparticles are considered: the gravitational force, $\vec{{\text{F}}_{\text{G}}}$ (Eq. 7, where $\vec{\text{g}}\left[m/s^{2}\right]$ is the gravitational vector); the drag force, $\vec{{\text{F}}_{\text{D}}}$ (Eq. 8, where ${\text{C}}_{\text{D}}$ is the drag coefficient, $\vec{{\text{u}}_{\text{p}}}\;\left[m/s\right]$ is the particle velocity vector, and ${\text{Re}}_{\text{p}}$ is the particle Reynolds number calculated according to Eq. 9); the pressure gradient force, $\vec{{\text{F}}_{\text{P}}\;}$ (Eq. 10); and the virtual mass force, $\vec{{\text{F}}_{\text{V}}\;}$ (Eq. 11, where ${\text{C}}_{V}$ is the virtual mass coefficient).

$$\vec{{\text{F}}_{\text{G}}}={\text{m}}_{\text{p}}\vec{\text{g}}\frac{\left({\text{ρ}}_{\text{p}}-\text{ρ}\right)}{{\text{ρ}}_{\text{p}}}$$ (7)

$$\vec{{\text{F}}_{\text{D}}}={\text{m}}_{\text{p}}\frac{18\text{μ}}{{\text{ρ}}_{\text{p}}{\text{d}}_{\text{p}}^{2}}\frac{{\text{C}}_{\text{D}}{\text{Re}}_{\text{p}}}{24}\left(\vec{\text{u}}-\vec{{\text{u}}_{\text{p}}}\right)$$ (8)

$${\text{Re}}_{\text{p}}=\frac{{\text{ρd}}_{\text{p}}\left|\vec{\text{u}}-\vec{{\text{u}}_{\text{p}}}\right|}{\text{μ}}$$ (9)

$$\vec{{\text{F}}_{\text{P}}\;}={\text{m}}_{\text{p}}\frac{\text{ρ}}{{\text{ρ}}_{\text{p}}}\vec{{\text{u}}_{\text{p}}}\left(\nabla \vec{\text{u}}\right)$$ (10)

$$\vec{{\text{F}}_{\text{V}}\;}={\text{C}}_{V}{\text{m}}_{\text{p}}\frac{\text{ρ}}{{\text{ρ}}_{\text{p}}}\left[\vec{{\text{u}}_{\text{p}}}\left(\nabla \vec{\text{u}}\right)-\frac{d\vec{{\text{u}}_{\text{p}}}}{dt}\right]$$ (11)

#### 2.2.2 Boundary Condition Methodology

##### 2.2.2.1 Inlet Boundary Conditions

**FIGURE 4:**
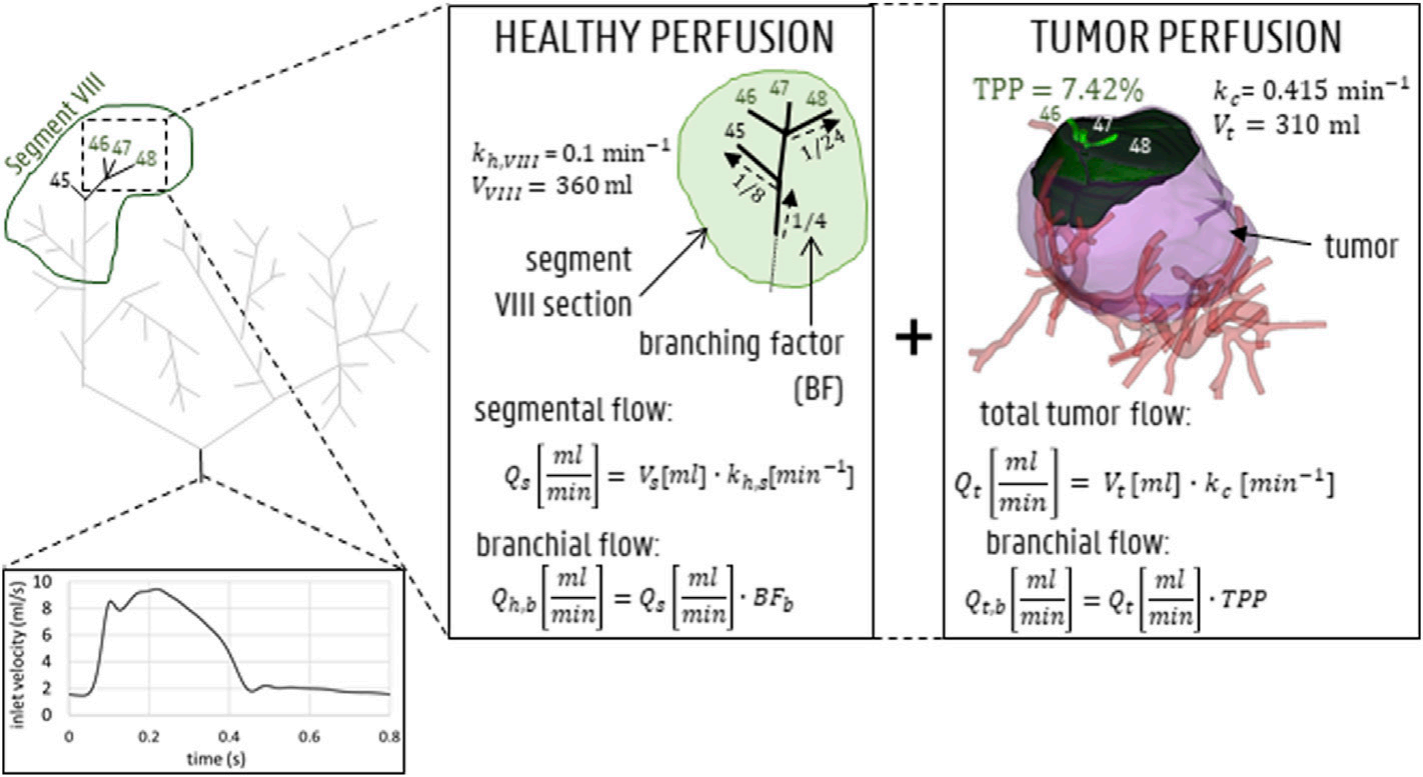
Inlet flow curve displayed for 1 representative cardiac cycle of 0.8 s. For the outlet boundary condition methodology, both healthy and tumoral flow contributions (which is 0 for outlets perfusing 0% of the tumor mass) are calculated for each outlet (shown here for outlets 46, 47, 48 in segment VIII).

For the inlet boundary conditions, a spatially uniform velocity profile was imposed, while the original geometry inlet was extruded by 80 mm as mentioned in Section 2.1.4 to account for the entrance length needed to let a more physiological flow profile develop. At the extruded inlet, a pulsatile waveform with a period of 0.8 s and a minimum/mean/max inflow velocity of 0.041/0.121/0.260 m/s was prescribed (see Figure 4). The waveform was derived from an in-house 1D model of the arterial circulation in humans (Campos Arias et al., 2017) and scaled so that the average inflow equaled the inflow as determined by the outlet boundary conditions (see below). Particles were injected every 0.01 s throughout the third cycle (allowing two prior cycles for flow development). For the planar injections (Sim. 1–3), particles were injected over the entirety of the axial injection plane (see Figure 3A). For the three catheter injections (Sim. 4–12), particles were injected (together with blood) at the start of the catheter at the hepatic arterial inlet corresponding to three different cross-sectional catheter tip locations (see Figure 3B). The particle injection velocity was set at 0.12 m/s (which corresponds with the mean blood flow velocity around the catheter tip), leading to a catheter flow rate of 2.77 ml/min.

##### 2.2.2.2 Outlet Boundary Conditions

Since the tumoral mass was mainly peripherally vascularized, it was considered likely that the arterial feeders of the tumor also, partly, perfused the surrounding healthy parenchyma. Therefore, the outflow of each outlet (denoted here as *b*) of Geometry 1, $Q_{b}\left[\frac{ml}{min}\right]$ , was considered as the summation of two flow terms: the healthy flow contribution, $Q_{h,b}\left[\frac{ml}{min}\right]$ , and the tumoral flow contribution, $Q_{t,b}\left[\frac{ml}{min}\right]$ . The healthy flow contribution for each outlet *b* of Geometry 1, $Q_{h,b}$ , was determined according to the methodology devised by Aramburu et al. (2016c). First, the volumes of each of the eight hepatic segments, $V_{s}\left[ml\right]$ , were set by scaling the literature-based segmental volumes to match the total liver volume of this patient-specific case (which was estimated as 1,357 ml in Mimics). Second, the total segmental arterial flow perfusing each segment *s*, $Q_{s}\left[\frac{ml}{min}\right]$ , was defined as:

$$Q_{s}=\;V_{s}\cdot k_{h\;}$$ (12)

where $k_{h}$ $\left[min^{-1}\right]\;$ is the healthy perfusion parameter, which equals 0.100 $min^{-1}$ (considered the same for all segments and determined by Aramburu et al. (2016c)). Third, the total segmental flow was divided over the different outlets perfusing one segment. From the CT-scans and the 3D model, it was derived which of the 48 outlets of Geometry 1 perfused which hepatic segment. Assuming that, within a segment, the flow split occurs symmetrically along each bi- or trifurcation, the healthy flow contribution of one arterial outlet, $Q_{h,b}$ , could be determined from the segmental flow, $Q_{s}$ by considering the intrasegmental branching fraction $BF_{b}$ (i.e., ½ for the first bifurcation, ¼ for the second bifurcation, etc.).

$$Q_{h,b}=Q_{s}\cdot BF_{b}$$ (13)

Concurrently, the tumoral flow contribution for outlet *b*, $Q_{t,b}\;\left[\frac{ml}{min}\right]$ , can be directly determined from the TPP of the region growing model. Similar to the methodology above, the total tumoral flow, $Q_{t}\left[\frac{ml}{min}\right]$ , can be calculated from the perfusion parameter for cancerous tissue, $k_{c\;}\left[\min^{-1}\right]$ , and the tumoral volume, $V_{t}\left[ml\right]$ :

$$Q_{t}=\;V_{t}\cdot k_{c}$$ (14)

Aramburu et al. previously theorized that, since the metabolic demand of tumor tissue is typically higher than that of healthy tissue, this should be reflected in the perfusion parameter, *k*. Here, $k_{c\;}$ was set equal to $0.415\;min^{-1}$ , over four times higher than the healthy perfusion parameter, $k_{h}$ (as previously estimated by Aramburu et al. (2016c)). Next, since the TPP reflects the fraction of tumoral volume that was perfused by one outlet, multiplying the TPP [%] with the total tumoral flow, $Q_{t}$ , gives the tumoral flow contribution of each branch.

$$Q_{t,b}=Q_{t}\cdot TPP$$ (15)

Finally, as explained before, the total outflow in branch *b* is the summation of the healthy and tumoral flow (which equals zero for healthy-perfusing branches) terms:

$$Q_{b}=Q_{h,b}+Q_{t,b}$$ (16)

The outflow boundary condition methodology is also shown in Figure 4 (for outlets 46, 47 and 48). In Table 2, the flow fractions (calculated according to the methodology outlined above) are given for each outlet in Geometry 1. Once $Q_{b}$ is determined for all outlets of Geometry 1, the total inflow through the inlet (through the principle of mass conservation) is also fixed. The healthy inflow contribution was 136 ml/min, while the tumoral inflow contribution was 129 ml/min, giving a total hepatic arterial inflow of 264 ml/min. This value was used to appropriately scale the inflow waveform, which was determined previously from the 1D model. The 1D inlet velocity waveform for 1 cardiac cycle is also shown in Figure 4. At the walls, the “no-slip” boundary condition was employed for the fluid phase. For the microparticles, the tangential and normal wall restitution coefficients were set to 1.

#### 2.2.3 Solver Settings

For pressure-velocity coupling, the SIMPLE algorithm was used; for spatial discretization, the gradient least-squares cell-based scheme was used; for pressure and momentum the second-order and second-order upwind schemes were used, respectively. The under-relaxation factors were kept at default (0.3 for pressure, 0.7 for momentum, 1 for density and body forces). The solution was initialized using a hybrid initialization scheme of 10 iterations. The time step size was varied between 0.5·10^−3^ s (for the acceleration and decelerating part of the cycle) and 1·10^−3^ s (for the flatter parts of the cycle). The maximum number of iterations specified for each time step was 50. Absolute globally scaled residuals lower than 1·10^−5^ were attained during every time step. Importantly, the particle distribution is sensitive to the total computational time: the more flow cycles are run, the more particles will exit the domain. To decide on the limit between convergence of the particle exit fractions and unnecessary computational time, additional flow cycles were run until <1.5% of the total injected particles exited in the latest cycle (leading to a range of simulations running for 9–14 cycles).

### 2.3 Post-Processing

#### 2.3.1 Particle Grid Methodology

**FIGURE 5:**
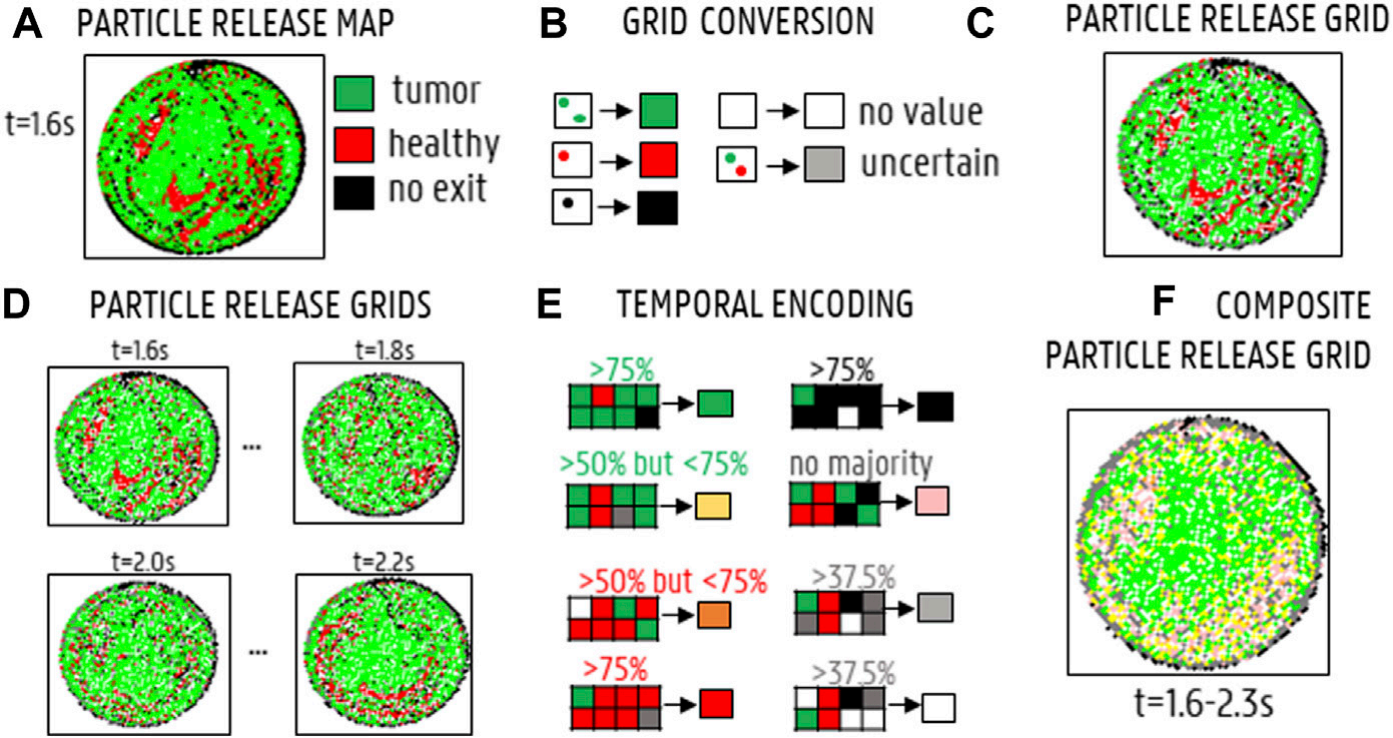
Generating Composite Particle Release Grids (CPRGs): **(A)** Particle Release Maps show the relation between injection location and particle fate (green: tumor, black: no exit, red: healthy, grey: spatially uncertain, white: no value). **(B)** By defining a reference grid and assigning each grid cell a color according to the particle fate of the injection positions within that cell, a Particle Release Grid (PRG) is obtained. **(C)** PRG for injection at the start of the cycle (“1.6s”). **(D)** PRGs visualized for four injection timings (“1.6s”,“1.8s”,“2.0s”,“2.2s′”). **(E)** By comparing PRG cell values for different injection timings, a CPRG is generated (green: “tumor” for >75% of the timings, red: “healthy” for >75% of the timings, yellow: “tumor” >50%, orange: “healthy” >50%, black: >75% “no exit”, pink: no majority fate found, grey: >37.5% “uncertain”, white: >37.5% “no value”). **(F)** CPRG generated for the full cycle, based on 8 injection timings (1.6–2.3 s, with a spacing of 0.1 s).

Particle Release Maps (PRMs) are typically used to visualize the impact of the cross-sectional injection location (for a given axial plane) on particle fate for a specific injection timing. Combining PRMs of multiple injection timings to visualize the impact of the cross-sectional injection location on particle fate throughout the cardiac cycle yields the Composite Particle Release Maps (CPRMs, as introduced by Childress et al. (2012) for simplified arterial geometries). Previously, Childress and Kleinstreuer (2014) also plotted PRMs against background reference grids to calculate the number of matching subsections between different PRMs (again for simplified geometries). Here, we use uniform reference grids to systematically replot the PRMs and call this the “Particle Grid methodology”, allowing to compute Particle Release Grids (PRGs) and Composite Particle Release Grids (CPRGs). The added value of the Particle Grid methodology is that comparing PRMs for different axial planes is difficult, because the density of the plane points may vary between different injections, which may result in unequal comparisons. This is not the case for PRGs, due to the use of the reference grid. In our Particle Grid methodology for patient-specific arterial geometries, CPRGs are generated in three succinct steps: 1) generation of Particle Release Maps (PRMs; as previously described and used in Bomberna et al. (2021)) spatially encoding these PRMs into Particle Release Grids (PRGs) by uniform resampling inside a two-dimensional plane, and 3) combining information of different PRGs, generated at different injection timings, into one CPRG representing the full cardiac cycle. First, Particle Release Maps (PRMs) are generated as color-coded visualizations of the axial injection plane at a specific injection timing, showing through which outlet a particle, injected at a specific cross-sectional location, exits (see Figure 5A for an example). As defined in Figure 5B, injection positions leading to particles exiting through one of the main arterial feeders of the tumor are annotated in green (“tumor”); injection positions leading to particles exiting through one of the healthy-perfusing branches are annotated in red (“healthy”); and injection positions leading to particles getting stuck and not exiting the domain are annotated in black (“no exit”). Next, Particle Release Grids (PRGs) are generated by plotting PRMs on a two-dimensional reference grid of equally-sized cells with a spacing of 1·10^−4^ m (Figure 5C). For each grid cell, only particle injection positions within the cell limits are considered: if all injected particles within that cell exit through tumor-perfusing branches, the cell value is denoted as “tumor” (colored in green); if all injected particles exit through healthy-perfusing branches, the cell value is denoted as “healthy” (red); if all injected particles remain stuck in the domain, then the cell value is defined as “no exit” (black); if some injected particles exit through tumor-perfusing branches and others through healthy-perfusing branches (or not exiting at all), the cell value is denoted as “spatially uncertain” (grey); if no particles were injected, the grid cell is denoted as “no value” (white) (Figure 5B). Note that the grid spacing must be patient-specific to balance the number of grey and white cells, as a grid spacing that is too large will result in mostly grey cells, and a grid spacing that is too small will result in a large number of white cells. For time-dependent inflows, multiple injection bursts occur throughout the injection cycle at specified intervals, and a PRG can be generated for each injection burst (see Figure 5D for a selection of PRGs at four injection timings). Since the spatial grids are identical for each burst, PRG cell values can be compared across different injection timings, resulting in the CPRG cell value. For this study, the CPRGs were composed based on eight selected injection timings with a spacing of 0.1s (1.6–2.3s) to represent temporal variation during the full cardiac cycle. The value of the CPRG cells is defined as follows (Figure 5E): if most PRG cells of the selected timings (>75%) are “tumor” (green), the CPRG cell value is denoted as “tumor/constant” (green); if most PRG cells (>75%) are “healthy” (red), the CPRG cell value is denoted as “healthy/constant” (red); if most PRG cells (>75%) are “no exit” (black), the CPRG cell value is denoted as “no exit/constant” (black); if <75% but >50% of PRG cells throughout injection is “tumor”, the cell value is denoted as “tumor/mostly” (yellow); if <75% but >50% of PRG cells is “healthy”, the cell value is denoted as “healthy/mostly” (orange); if PRG cells are divided between “tumor”, “healthy” and “no exit” without any of the above rules applying, then the resulting value is “temporally uncertain” (pink); however, if PRG cells are divided, but >37.5% of cells are “no value” (white), then the CPRG cell is denoted as “no value” (white); and similarly, if >37.5% of the cells are “spatially uncertain” (grey), the CPRG cell is denoted as “spatially uncertain” (grey), as well. As a result, merging multiple PRGs into a Composite Particle Release Grid (CPRG) combines spatial and temporal information on particle fate (Figure 5F).

#### 2.3.2 Grid-Based Particle Distribution

For the planar injections, the PRGs for all injection timings throughout the injection cycle can be used to determine the grid-based particle distribution. For each outlet *x*, the “cell fraction” (or CF_x_) can be determined, which is the number of grid cells associated with outlet *x* divided by the sum of the number of cells associated with one of the 48 outlets and the “no exit” outlets (Eq. 17).

$$CF_{x}=\frac{\#cell_{x}}{\sum_{x=1}^{48}\left(\#cell_{x}\right)+\#cell_{no\;exit}}$$ (17)

For Geometries 2 and 3, the CF can only be calculated for the 38 and 17 outlets of those geometries, respectively. Therefore, to determine the particle distribution at all 48 outlets of the original geometry, it is assumed that, beyond the truncated outlets of Geometry 2 and 3, particles distribute themselves proportional to the imposed flow distribution of Geometry 1. The resulting model is a hybrid particle-flow model, where particle distribution is modelled until the level of the truncated outlets, and the remainder of the particle distribution is modelled by the flow distribution. As a result, the CFs for Geometry 2 and 3 are not technically the same “cell fractions” as for Geometry 1; in these truncated geometries, they are named the “truncated cell fraction” (or TCF), and can be calculated as:

$$TCF_{x}=CF_{x*}\cdot FF_{x}$$ (18)

where FF_x_ is the flow fraction of outlet *x* ( $Q_{x}$ in Eq. 19), i.e., the outflow BC of outlet *x* in Geometry 1 divided by the outflow BC of the upstream, truncated outlet x* in Geometry 2 or 3 ( $Q_{x*}$ in Eq. 19). As a simple example, if a trifurcation of equal-flow branches (*x*_*1*_ *-x*_*3*_) in Geometry 1 is simplified into a truncated single branch (x*) in Geometry 2, the outflow BC in the truncated single branch *x∗* will be three times the flow in any of the original trifurcation branches *x*_*1*_ *-x*_*3*_; consequently, FF_x_ will be 1/3, and particles exiting outlet *x∗* in Geometry 2 will be assumed to distribute evenly among branches *x*_*1*_ *-x*_*3*_). Hence, FF_x_ can simply be determined as:

$$FF_{x}=\frac{Q_{x}}{Q_{x*}}$$ (19)

#### 2.3.3 Monte Carlo-Based Tumor Dose Distribution

The (T)CF_x_ can be calculated for the full cross-section, but also for the small section of the grid (i.e., the axial injection plane) that would coincide with the catheter tip exit for a catheter injection (see Figure 3 for the annotated catheter tip locations). This catheter-associated (T)CF_x_ offers an estimation of the particle distribution after catheter injection for a hypothetical catheter injection location. The tumor dose (TD in Eq. 20) can then be estimated by multiplying the (T)CF_x_ with the tumor flow contribution for outlet *x* and adding together these contributions for all outlets. This gives a measure of the total number of particles flowing to the tumor (i.e., total “dose”):

$$TD=\sum_{all\;outlets\;\text{x}}\left(T\right)CF_{x}\cdot \frac{Q_{t,x}}{Q_{t,x}+Q_{h,x}}$$ (20)

When the catheter-associated grid section is shifted randomly across the injection plane to represent randomly sampled catheter injection locations, the tumor dose distribution shows how much the tumor dose changes for random injection locations within the injection plane. Essentially, this tumor dose distribution can offer a direct quantification of the differences between the different (C)PRGs obtained for the simulations as listed in Table 1, since similar (C)PRGs should lead to similar tumor doses (and tumor dose distributions). This methodology represents a Monte Carlo-based framework because the deterministic parameter of catheter injection location is treated stochastically (i.e., varying over the plane) (Johansen, 2010). Here, the sampling was done in Matlab (MathWorks, United States), with a uniform distribution for all grid cells included in the sampling set. However, grid cells that were located too close to the periphery (i.e., when catheter tip placement was not possible) were not deemed “appropriate” and excluded from the sampling set. Essentially, these were grid cells where a 7x7-square (i.e., with sides of 7·10^−4^ m) could not be placed around the central cell.

#### 2.3.4 Catheter Particle Distribution

For the catheter injections in Geometry 1, the particle distribution (also deemed the “exit fraction” (EF_x_) in Eq. 21) can simply be calculated as the fraction of the number of particles exiting through outlet *x* over the total number of particles which exit the catheter (Eq. 21). Again, the truncated EFs (TEFs) are calculated by considering that the particles exiting the outlets in Geometry 2 and 3 distribute proportionally to the flow distribution (Eq. 22), with FF_x_ defined as before.

$$EF_{x}=\frac{\#particles_{x}}{\#particles_{catheter-exit}}$$ (21)

$$TEF_{x}=EF_{x*}\cdot FF_{x}$$ (22)

## 3 Results

To determine the impact of geometry truncation on the particle distribution, the planar and catheter injections in each of the three geometries are compared. First, the CPRGs are compared in Section 3.2.1, both visually and quantitatively (based on the TD distribution for a number of randomly sampled catheter injection locations). Second, the grid-based particle distribution (i.e., (T)CFs) for the planar injections (Section 3.2.2) and the (T)EFs resulting from catheter injections (Section 3.3) are compared in each corresponding geometry.

### 3.1 Particle Progression in Domain

**FIGURE 6:**
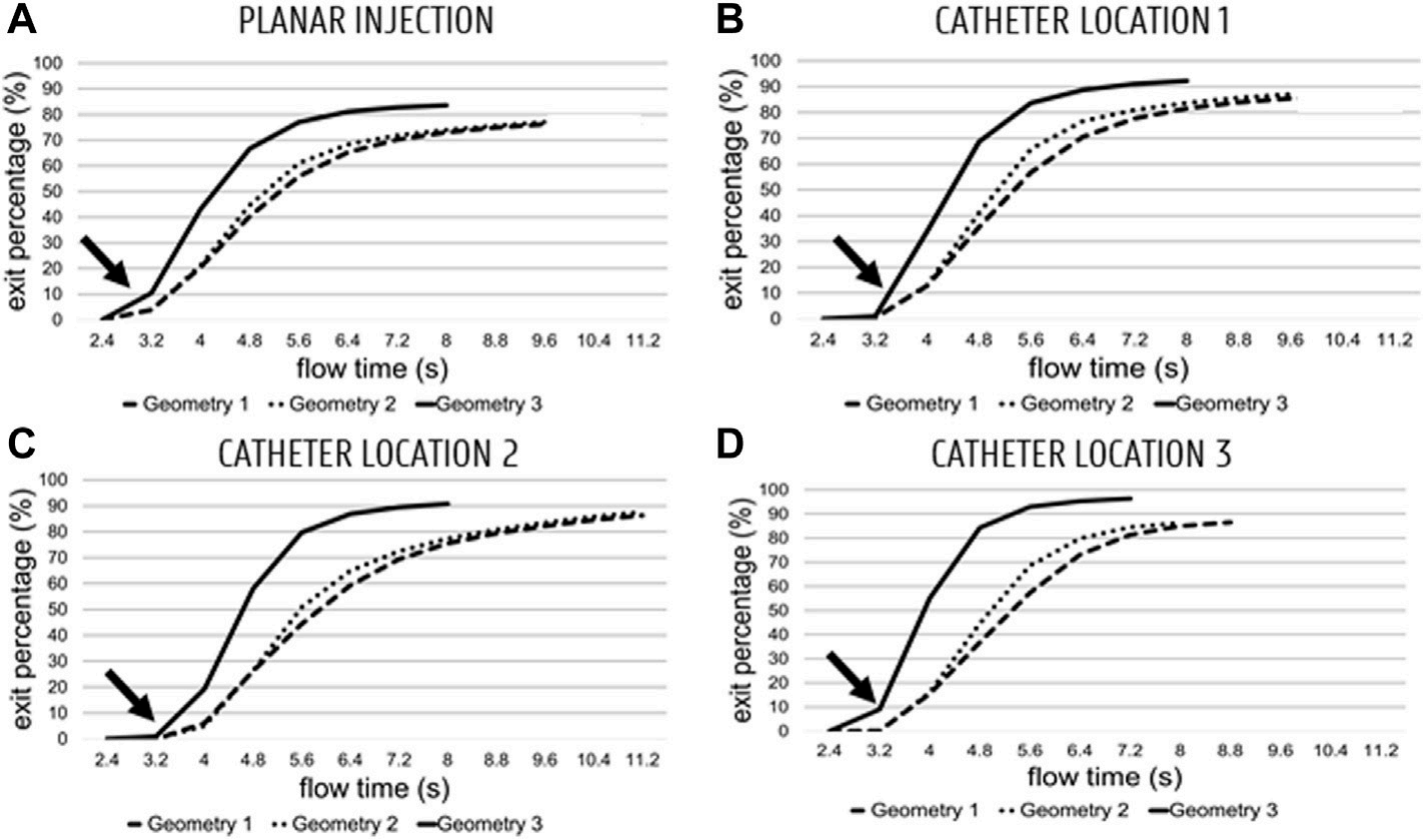
Cumulative particle exit fractions for Geometries 1–3 (Geometry 3 is the top curve, Geometry 1–2 are the dashed curves) plotted against flow time (plotted from the start of the fourth flow cycle onwards; 2.4 s). **(A)** Planar injections. **(B–C–D)** catheter locations 1, 2 and 3, respectively. Globally, particles start exiting starting at the start of the 5th cycle (3.2 s—see arrow).

The cumulative particle exit fraction (relative to the total number of injected particles) is plotted in Figure 6. Generally, it can be seen that the particles started exiting from the fifth flow cycle onwards (see t = 3.2s and arrows in Figure 6). For the planar injections (panel A in Figure 6, 12 flow cycles were needed for Geometry 1 (9 after the end of the particle injection cycle), 12 for Geometry 2, and 10 for Geometry 3. For the first catheter injection in Geometry 3 (panel B), only 10 cycles were run, compared to the 12 cycles necessary for Geometry 1 and 2. For the second catheter injection (panel C), 4 cycles less were needed for Geometry 3 than for Geometry 1 and 2 (10 compared to 14); for the third catheter injection (panels D), 2 cycles less were needed for Geometry 3 than for Geometry 1 (9 compared to 11), and 1 cycle less was needed for Geometry 2 compared to Geometry 1.

### 3.2 Planar Injections

#### 3.2.1 Particle Grids

**FIGURE 7:**
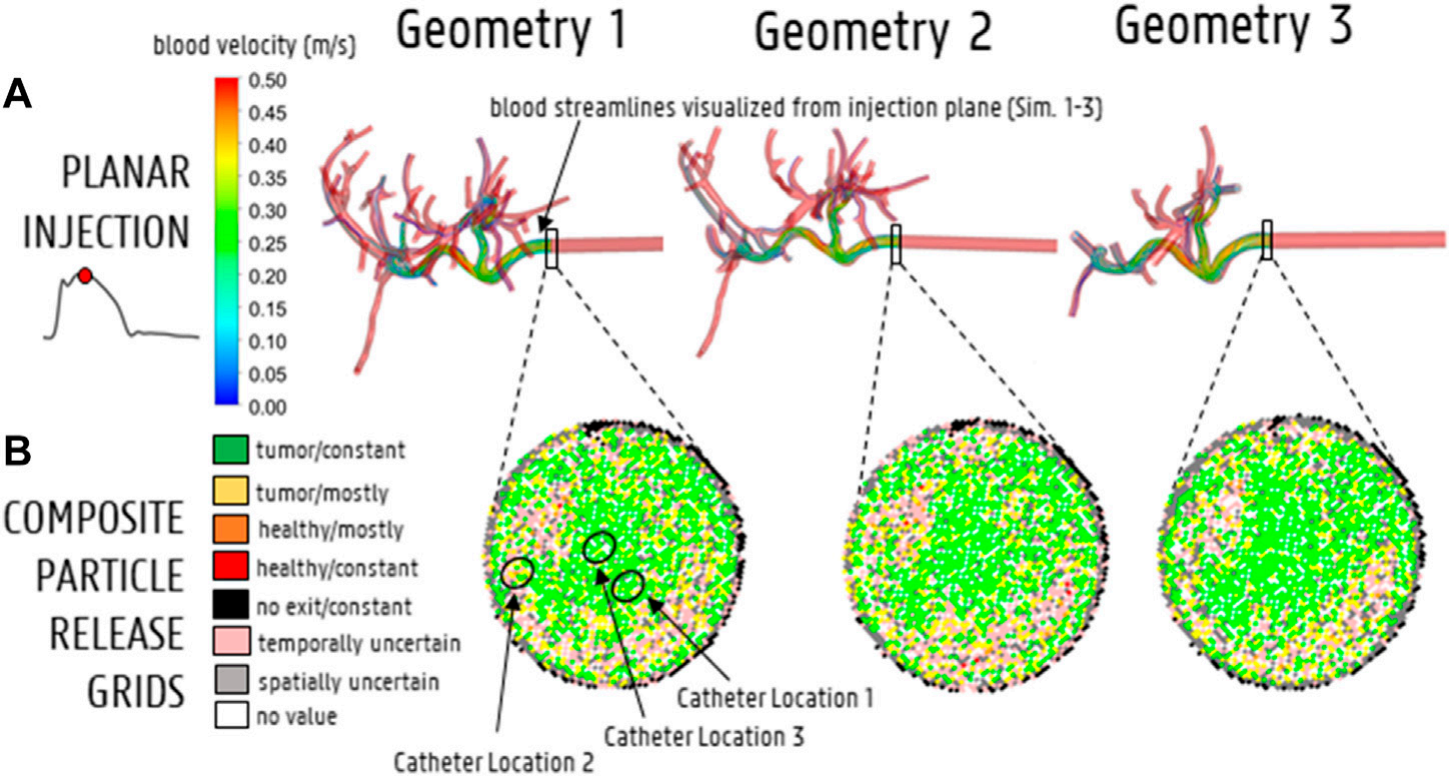
**(A)** Streamline visualization at peak systole for particle release at the injection plane for Geometry 1–3 (Sim. 1–3). **(B)** The CPRGs for the 3 geometries show that particles injected at the center of the cross-section (green zone) lead to >75% of tumor targeting throughout the cycle. Comparing CPRGs, consistency of the major visual trends across all geometries is visible, with only minor differences.

**FIGURE 8:**
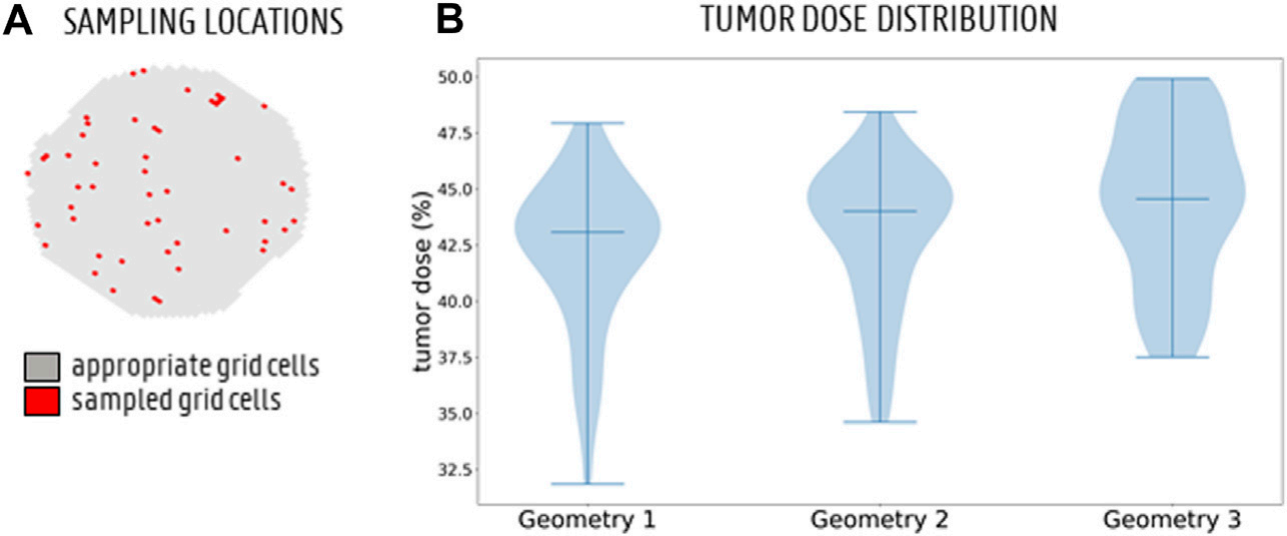
**(A)** The full sampling set of appropriate grid cells (grey) and 50 sampled grid cells (red) to simulate particle injection from these catheter tip locations. **(B)** Monte Carlo distribution of tumor dose (TD) for 50 randomly sampled catheter injection locations in each geometry. The 3 bars denote the minimum, median and maximum, respectively. The violin plot for Geometry 3 looks slightly different than for Geometries 1 and 2, but the medians are very similar.

For the planar injections, the CPRGs of the axial injection plane of each geometry are displayed in Figure 7. Visually, there is some mismatch between the three CPRGs, although the major trends are similar for all geometries. At the center of the CPRG, there is a large green zone. Peripherally, a U-shaped zone of pink and yellow cells appears. The border is dominated by black or grey cells, while there are no large zones of orange or red cells. To study the differences between PRGs quantitively, the TD distribution for 50 randomly sampled catheter injections in each geometry are given in a violin plot in Figure 8, with the colored area surrounding the boxplot representing the sample density. The ranges in TD are 31.9%–47.9% for Geometry 1, 34.6%–48.4% for Geometry 2, and 37.5%–49.9% for Geometry 3; the median TDs are 43.1, 44.0, and 44.6%, respectively.

#### 3.2.2 Grid-Based Particle Distribution

**FIGURE 9:**
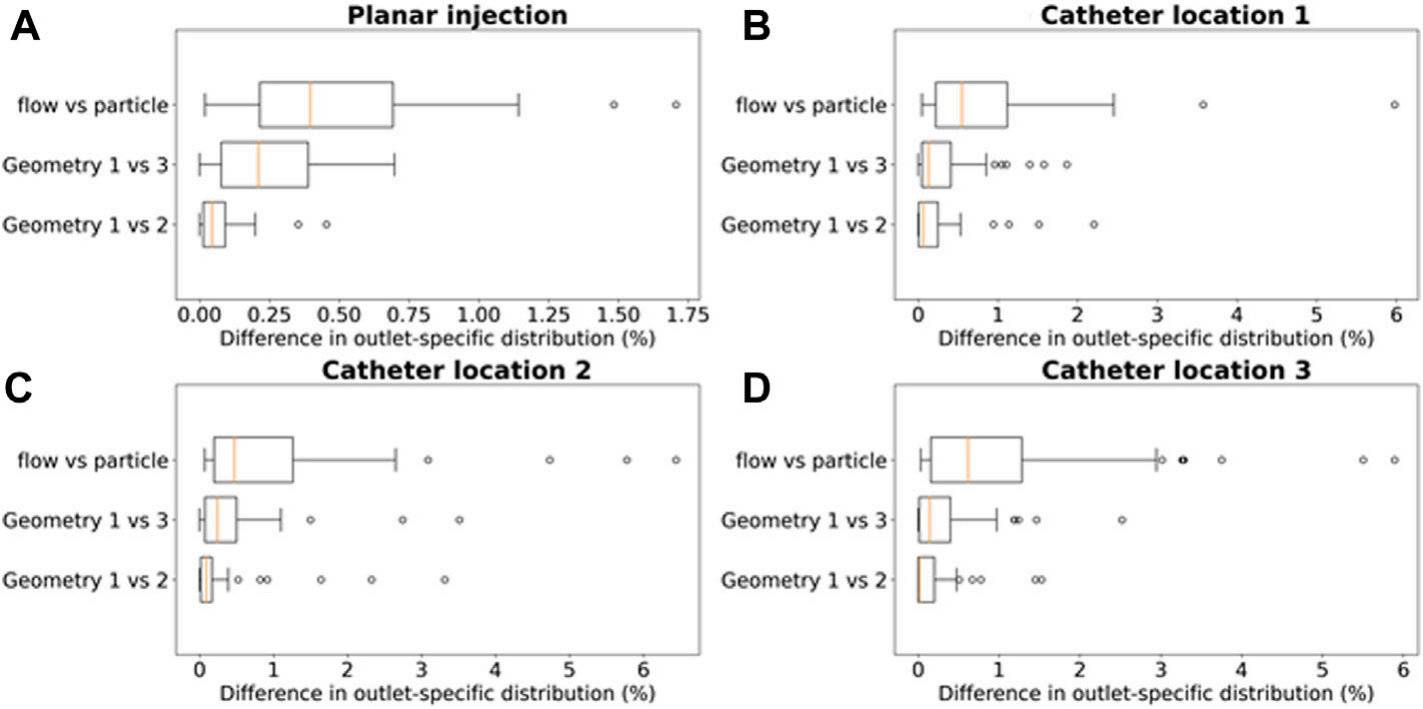
Boxplots comparing the differences in outlet particle distribution ((T)CF in panel A, (T)EF for panels **(B–D)** in Geometry 1 and 2 (“Geometry 1 vs. 2”), Geometry 1 and 3 (“Geometry 1 vs. 3”), and the differences in flow distribution and particle distribution in Geometry 1 (“flow vs. particle”). This is done for each of the 4 simulation sets: **(A)** the planar injection, and **(B–C–D)** catheter locations 1–3. For the planar injection, the error range is clearly much smaller than for the catheter injections (maximum of 1.75 vs. 6%).

Computing the grid-based particle distribution for the planar injection (Figure 9A), the minima, maxima, median and interquartile ranges of the absolute (T)CF differences between the geometries are reported in Figure 9 (“Geometry 1 vs. 2”: comparing particle distribution between Geometry 1 and 2, “Geometry 1 vs. 3”: comparing particle distribution between Geometry 1 and 3, “flow vs. particle: comparing flow and particle distribution in Geometry 1). The median difference in outlet-specific (T)CF between Geometry 1 and 2 is 0.04% (with a reported maximum of 0.45% in outlet 48). The median difference in outlet-specific (T)CF between Geometry 1 and Geometry 3 is 0.21% (with a maximum of 0.70% reported in the truncated outlet 42). Comparing the flow distribution and the CF in Geometry 1, the median outlet-specific difference is 0.40% (maximum of 1.71% reported in outlet 36).

### 3.3 Catheter Injections

**FIGURE 10:**
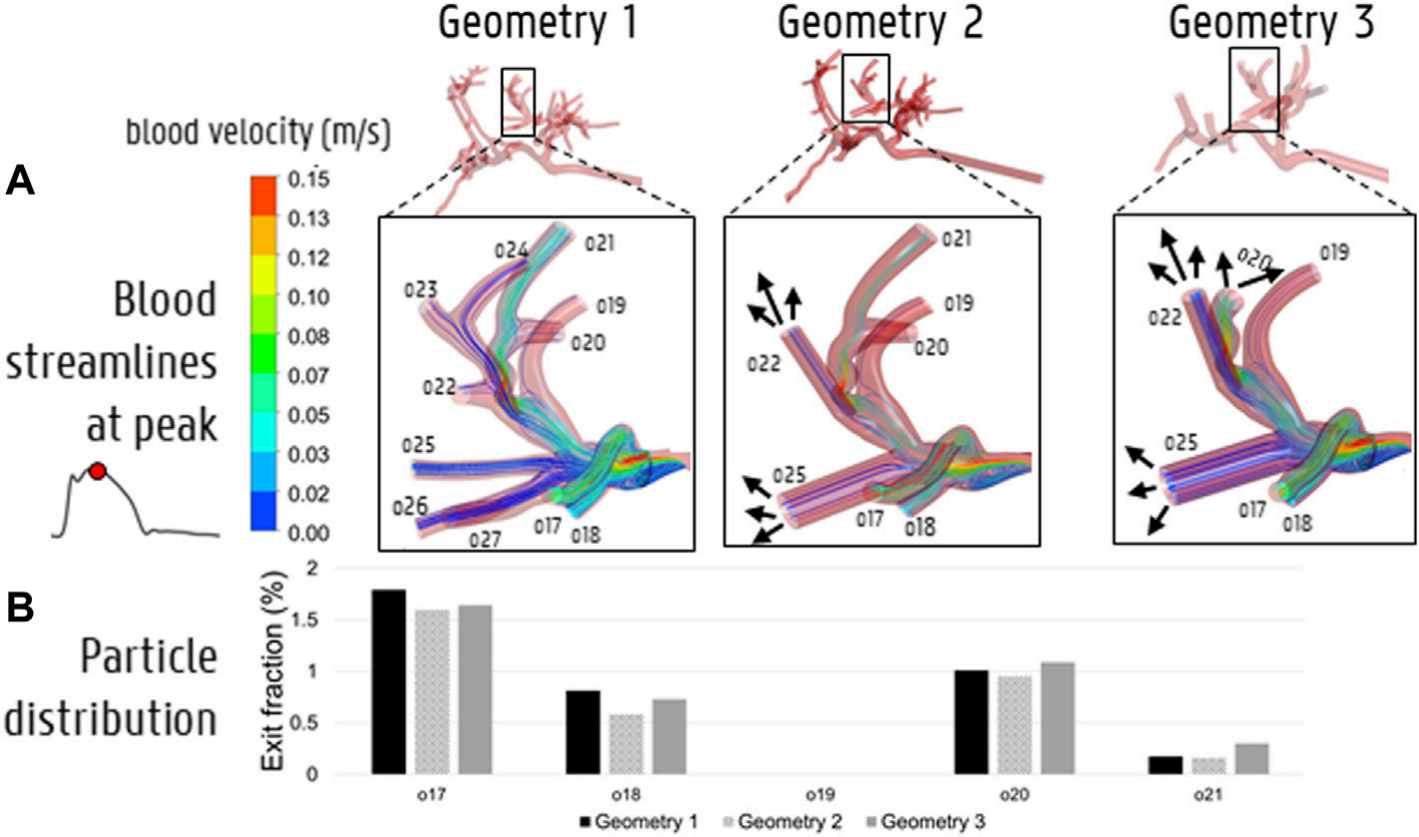
**(A)** The impact of simplifying the hepatic arterial geometry (Geometry 1–2–3) on the blood streamlines at peak systole. The arrows indicate where outlets were truncated with respect to Geometry 1, i.e., where the downstream flow distribution was used to model the particle distribution beyond the level of this truncated outlet. **(B)** The impact of truncation on particle distribution in outlets 17–21.

To compare the particle (and flow) distribution after the catheter injections the three geometries, the median, maximum, interquartile ranges and outliers of the absolute (T)EF differences between the three geometries are displayed in Figures 9B-C-D. With regards to the (T)EF per outlet for the first catheter injection location (see Figure 3B), the median difference between Geometry 1 and 2 is 0.06% (maximum of 2.20% reported in outlet 28). The median difference between Geometry 1 and 3 is 0.13% (maximum of 1.86% reported in outlet 29). In Figure 10, the streamlines at peak systole during the particle injection cycle (cycle 3) are shown in several of these truncated outlets and compared to the hemodynamics in the original outlets, highlighting the impact of geometry on blood flow for both planar and catheter injections. The arrows in the panels for Geometry 2 and 3 indicate where outlets were truncated (and thus, where particle distribution was estimated based on solely flow modeling). For outlets 17–21 in Geometry 1, the EFs after catheter injection at Location 1 (Sim. 4) are compared to the TEFs of Geometry 2–3. The (T)EFs for the remaining outlets 22–27 were not compared visually in Figure 10B because the difference between geometries were negligible (<0.10% for each outlet). For the second injection location, the median difference in (T)EFs between Geometry 1 and 2 is 0.09% (maximum of 3.32% in outlet 17) and 0.24% between Geometry 1 and 3 (maximum of 3.51% in outlet 17). For the third injection location, the median difference in (T)EFs between Geometry 1 and 2 is 0.02% (maximum of 1.53% in outlet 42); the median difference between Geometry 1 and 3 is 0.14% (maximum of 2.52% in outlet 44). Next, the particle EF and flow distribution in Geometry 1 are compared. The median differences between outlet-specific EFs and outflows for the three catheter locations are 0.55, 0.24, and 0.62%, respectively. The maximal outlet-specific EF and outflow differences reported for these catheter injections are 5.97, 6.44, and 5.89%, respectively.

## 4 Discussion

With regards to the computational strategy for determining the particle distribution in the patient-specific hepatic arterial geometry, four approaches can be outlined: 1) modeling the full-complexity particle distribution in Geometry 1; 2) using the hybrid particle-flow model in the simplified Geometry 2, or 3) in the much more simplified Geometry 3; and, 4) assuming that the flow distribution is an appropriate estimator of the particle distribution. In approaches (i)-(iii), the microparticle behavior is explicitly modelled (until a certain level); while, in approach (iv), it is not. Importantly, each approach impacts the total computational complexity. Below, the accuracy of the obtained results of each approach are discussed (compared to the baseline particle distribution in Geometry 1, i.e., approach (i)).

### 4.1 Computational Cost

The plots of cumulative exit fraction with respect to flow time in Figures 6A,B show that the advantage of simplifying the hepatic arterial geometry from Geometry 1 to Geometry 3 with respect to computational cost and time is twofold. First, the mesh contains significantly less mesh elements, so overall computational cost decreases. Second, more particles also exit the truncated Geometry 3, and less flow cycles are needed to reach particle exit convergence. By truncating Geometry 1 to Geometry 2, these advantages are more limited: the decrease in mesh elements is not so significant (for Catheter Location 3 even non-existent), and the same number of flow cycles is needed to reach convergence (with the exception of Catheter Location 3). As an illustrative example of the impact of truncation on the computational time (determined here explicitly for Catheter Location 1), the average flow cycle time was 65 min for Geometry 1, 61.75 min for Geometry 2 and 57.25 min for Geometry 3 (run on a High-Performance Computing cluster with 384 cores and 250 GB RAM). Thus, by only considering the decrease in mesh elements, the computational cost of Geometry 3 is ∼12% lower than for Geometry 1. However, since only 10 flow cycles were needed for Geometry 3 to reach particle exit convergence, while 12 flow cycles were needed for Geometry 1, the total estimated computational time was 780 min for Geometry 1 and 572.5 min for Geometry 3. This indicates a ∼27% decrease in total computational cost by truncating Geometry 1 to Geometry 3 for the full simulation.

### 4.2 Planar Injections

The CPRGs of the three geometries (Figure 7) show similar global trends: particles which are injected at the periphery of the cross-section have more trouble exiting the domain (black cells); the center of the cross-section is the ideal injection location to target the main tumor feeders of this liver (green cells); near the east to north-west periphery, the uncertainty of tumor targeting increases (U-shaped zone with increasing number of yellow or pink cells). There appear to be only a few cells where injection leads to steering particles away from the main arterial feeders (red or orange cells). Conceptually, this means that the central green zone would be the preferred injection location over the more uncertain, U-shaped zone. Visually, these major trends seem consistent between geometries, although some minor differences between the CPRGs are apparent. Studying the Monte Carlo-based TD distributions, the sampled grid cells (the same for all three geometries) are given in Figure 8A. As is clear from Figure 8B, the overall TD range (minimum-maximum) is highest for Geometry 1 (spread of 16.1% instead of 13.8% for Geometry 2 and 12.4% for Geometry 3). In general, the TD distribution is slightly more concentrated for Geometry 3, while the bell-like distribution shapes for Geometry 1 and 2 are more similar to each other. This discrepancy for Geometry 3 is most likely due to the increased truncation level. However, the impact on the medians of the distribution is limited (43.1% for Geometry 1, 44% for Geometry 2, and 44.6% for Geometry 3). Importantly, this means that the predicted impact of fluctuations in catheter tip location on the tumor dose is slightly larger for Geometries 1 and 2 (i.e., larger ranges), while the overall expected tumor dose (i.e., mean of the TD distribution) is slightly higher for Geometry 3. However, differences are very limited, and it can be stated that the impact of truncation on the TD distributions is not significant. The grid-based particle distribution for the planar injections in Geometry 2-3 shows that the median differences in particle distribution with respect to Geometry 1 are very small (<0.25%) when truncating the geometry. As seen in Figure 9, the median difference increases slightly when truncating Geometry 2 to Geometry 3 (from 0.04 to 0.21%), illustrating the impact of truncation. Based on the limited median differences (<0.25%), the limited maximal differences (<0.70%) and the preservation of the major trends in the CPRGs, it can be said that the accuracy loss for a planar injection after truncating of Geometry 1 to Geometry 3 using the suggested pruning algorithm is limited for the patient-specific liver studied. When modeling only the flow distribution in the planar injection, the median and maximum differences between flow and particle distribution increase further (to 0.40 and 1.71%, respectively); indicating a decrease in accuracy that is caused by not modeling the particle distribution. However, due to limited maximum differences (<2%), it can be stated that, while modeling the particles has a clear advantage over modeling only the flow, the flow distribution is a decent estimator of the full-complexity particle distribution for the planar injection considered in this liver. If release maps similar to CPRGs need to be obtained, CFD simulations can be used to generate the flow pathlines after planar injection, as shown by Taebi et al. (2021). To estimate only the particle distribution, the CFD simulation would not even be needed, reducing the simulation time to 0 (Aramburu et al., 2022). However, it should also be emphasized that the particles in this study are small, and that fluid-particle differences may increase for larger particles (i.e., for TACE).

### 4.3 Catheter Injections

With regards to the differences in microparticle behavior for the three catheter injections (“Geometry 1 vs. 2” and “Geometry 1 vs. 3” in Figures 9B-C-D), the median differences in the outlet-based EF between Geometry 1 and 2 (<0.10%) and Geometry 1 and 3 (<0.30%) are still very small for each catheter injection separately. However, for the maximal outlet-specific differences, some higher outliers (∼3.50%) are reported than for the planar injections. Also, the median and 75th percentile of the difference in particle distribution are always larger for Geometry 3 than for Geometry 2 (although the maxima are of a similar order of magnitude), illustrating the impact of truncation. However, these differences are still minor, indicating that the particle distribution in Geometry 1 can be reliably estimated by the hybrid-particle flow model using the truncated Geometry 3 (at least for this patient-specific case). It is also clear from Figure 9 that for the first and third catheter injection (panels B–D), the hybrid particle-flow model using Geometry 3 offers a significantly better estimation of the full-complexity particle distribution than using just the flow distribution, given the significantly smaller median and maximum differences for the truncated particle distribution. For the second catheter injection (panel C), this is still the case, but the discrepancy is less clear: the median difference in EF between Geometry 1 and 3 (0.24%) is still less than the median difference in flow and particle distribution (0.47%), but not as significantly as for the other injection locations. When using just the flow distribution, maximal outlet-specific differences of ∼6% are reported, which are significantly higher than the outliers for the catheter injections (∼3.50%) and for the planar injection (∼2%). Especially considering that numerous outlets have low outflows (30/48 outlets in Geometry 1 have <2% imposed outflows; maximum imposed outflow is 13.3%), absolute outlet-specific differences of ∼6% are relatively high. These results show that using the flow distribution as a surrogate for the particle distribution is considerably less accurate than using the hybrid model, indicating that flow modeling is preferably combined together with explicit particle modeling in the first generations of the tree (at least for catheter injections).

### 4.4 Summary

**FIGURE 11:**
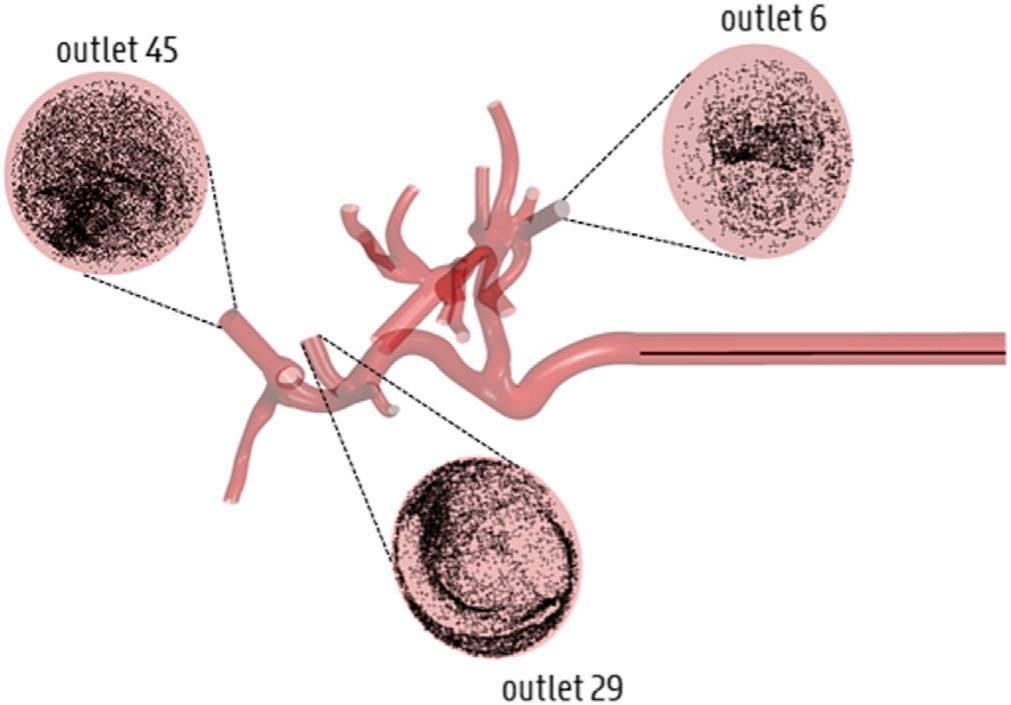
Illustration of the spread of particles across the cross-section by the time they reach the outlets of Geometry 3 (displayed here for Catheter Location 1).

Summarizing the results of this study, the accuracy loss of the estimated particle distribution by truncating Geometry 1 to Geometry 3 is limited, but higher than truncating from Geometry 1 to 2, indicating the impact of truncation. However, truncating Geometry 1 to Geometry 2 does not offer much added value, since the decrease in computational time is very limited; in that sense, truncating Geometry 1 to 3 offers much more added value due to the limited accuracy loss and higher decrease in computational time. Meanwhile, the accuracy loss in each geometry is significantly higher when only the flow distribution is modeled (compared to also explicitly modeling the particle distribution). However, using the flow distribution as a surrogate for particle distribution is justified for the planar injection due to limited accuracy loss, but less so for the catheter injections. This indicates the importance of explicitly modeling the particle distribution for catheter injections, at least to the level of Geometry 3. These findings make sense because particles follow a select number of blood streamlines after catheter ejection, and will not mimic the flow distribution initially. However, for planar injections, particles are spread over the arterial cross-section, and tend to mimic the flow distribution more. Figure 11 shows that particles, after catheter injection in Geometry 3, have spread out over the entire arterial cross-section by the time they reach the outlets (albeit non-uniformly). This could partly explain why explicit particle modeling beyond the level of the truncated outlets of Geometry 3 is not strictly needed, and why hybrid particle-flow modeling may approximate the full-complexity particle distribution well enough (at least for this patient-specific geometry).

### 4.5 Limitations and Future Work

This study puts forward an important approach to decrease computational complexity for current CFD simulations of transarterial radioembolization for HCC. We introduced a hybrid particle-flow model, as the results of this study stress the importance of modeling particle behavior in the first few generations of the hepatic arterial geometry (especially for catheter injections), but at the same time allowing to truncate the geometry further downstream according to the presented truncation algorithm. The additional novelty of this work lies in the consideration of a novel region growing method to determine outflow boundary conditions; modeling of outflow boundary conditions for outlets that perfuse both healthy and tumor tissue; and generating patient-specific CPRGs, which had never been done before (neither for CPRMs). Importantly, this study also has several limitations. With regards to the modeling approach, only straight catheters were studied. The catheter itself was modelled as thin-walled, and the perfusion fluid inside the catheter lumen, which is typically saline, was modelled as blood. The outlet boundary conditions were considered as outlets with constant outflow fractions; realistically, these outflows can vary as the treatment procedure carries on, as distal penetration of the microspheres might obstruct or even fully occlude downstream vessels, and increase the flow resistance. This effect could be even more considerable when modeling chemoembolization procedures. Concurrently, the tumor mass was considered peripherally vascularized, and it was not considered that the tumor might be multilobed, with regions that are both peripherally and internally vascularized. Considering the inflow boundary conditions, the inlet was artificially extruded to obtain a fully developed flow profile, which was symmetric by design; however, in reality, it is possible that the inlet flow profile is skewed, which might impact the (C)PRGs and downstream particle distribution. Therefore, patient-specific measurements (like 4D-flow MRI) could be used to determine patient-specific inflow conditions. Finally, only one particle type was modelled here, while many different particles types with distinct biophysical properties are commercially available (i.e., larger particles, which might decrease the accordance between flow and particle distribution and increase the importance of explicit particle modeling). With regard to future work, the Monte Carlo-based tumor dose distribution cannot only be used to compare (C)PRGs, but also to quantify the impact of the catheter tip location for given (C)PRGs. Specifically, a high range in tumor dose distribution indicates large variations within the (C)PRG, which may indicate that small fluctuations of the in-plane catheter tip location can lead to significantly different tumor doses. This is crucial, since it is currently technically unfeasible to accurately control the catheter tip location *in vivo*, and determining the tumor dose distribution for a given axial plane may help to quantify the uncertainty regarding catheter tip location for that plane. In that sense, the median of the tumor dose distribution can be considered the “expected” tumor dose, given the range of uncertainty due to possible fluctuations in catheter-tip location. The “expected” tumor dose can be compared for both proximal and distal injection locations to determine which injection type would be more likely to be clinically successful. Furthermore, the appropriate truncation level should be validated for more distal catheter injection locations, as it would likely take particles longer to spread over the arterial cross-section (see Figure 11) when injected at more downstream axial injection locations. Next, the boundary condition methodology based on the tumor region model should be experimentally validated and region growing could be expanded to include the entire liver. In that case, internally and peripherally vascularized tumor regions could be distinguished by comparing the sizes of the healthy parenchyma and tumoral volumes of each branch against each other (i.e., for internally vascularizing branches the perfused tumoral volume should be much larger than the perfused healthy volume). Importantly, all findings reported here should be interpreted with respect to this patient-specific case, and cannot simply be extrapolated to other patients. In the future, more patient-specific cases should be considered with diverse scenarios of cancer involvement (e.g., cases with multiple small tumor nodules), and aim to replicate the findings discussed in detail here. In summary, this work introduces a hybrid particle-flow model for truncated arterial trees and evaluates the suitability of this model for a patient-specific HCC case. This alternative approach to CFD modeling of radioembolization of liver tumors should allow to decrease the computational cost of future CFD simulations.

## Data Availability

The raw data supporting the conclusions of this article will be made available by the authors, without undue reservation.

## Abbreviations

HCC, hepatocellular carcinoma; TACE, transarterial chemoembolization; TARE, transarterial radioembolization; Tc-99m-MAA, Technetium-99m-macroaggregated albumin; CFD, computational fluid dynamics; TPP, tumor perfusion percentage; (C)PRM, (Composite) Particle Release Map; (C)PRG, (Composite) Particle Release Grid; (T)CF, (truncated) cell fraction; (T)EF, (truncated) exit fraction; TD, tumor dose.
